# Nasal Catarrh

**Published:** 1885-10-20

**Authors:** John A. Henning


### NASAL CATARRH.

By JOHN A. HENNING, M. D.

Here in Kansas, and through the Western States, we have a large number of cases of nasal catarrh. Whether it is caused by sudden change of the weather, or some kind of vegetable parasite or irritant, I am unable to determine. However, I am inclined to believe the latter. But let the cause be what it may, the disease in question is quite prevalent among us, and it is our duty to cure it.

Nasal catarrh is both acute and chronic, and may be mild or severe in character. In the acute form it is frequently ushered in with slightly chilly sensations, slight headache above one or both eyes, frequent sneezing, the mucous membrane of the nerves becomes red and swollen, and there is a glary white mucus discharge from the nostrils. The appetite is indifferent, bowels costive, and the patient is unable to perform manual labor. These

---

*Versuch einer Bacteriotherapie. Centralblatt fur die Medicinischen Wissenschaften, No. 2., July 18, 1885.

are the most prominent symptoms in the acute form, and will last two or three weeks, and then, unless arrested, will run into the chronic form, which may continue for years. But the chronic form is hardly ever stationary; in the course of time it will invade the eustachian tubes, lungs, and finally in some cases terminate in catarrhal phthisis pulmonalis.

TREATMENT.

I have treated hundreds of cases of nasal catarrh in all its various forms and stages, and have tried all kinds of remedies and instruments, and after close observation, have settled upon the following course of treatment:

℞. Arsenici iodidi............................gr. viij
Aquæ dest................................ O j

M.—Sig. Take a teaspoonful three times a day. Continue the remedy for months, in either acute or chronic form.

Also:

℞. Potass. permanganas.........................gr. iv
Aquæ dest ....... ..............................℥ iv

M.—Sig. Pour a small quantity in the palm of the hand and snuff up each nostril sufficiently hard so that the solution will run through the nostrils and reach the pharynx. Repeat night and morning.

This treatment cures a large majority of cases speedily and permanently. I have abandoned all forms of instrument treatment and all but the above, and can heartily recommend it to the profession.—*Medical World.*

